# Investigation of Exfoliation Efficiency of 6H-SiC Implanted Sequentially with He^+^ and H_2_^+^ Ions

**DOI:** 10.3390/ma15082941

**Published:** 2022-04-18

**Authors:** Guoqiang You, Haipeng Lin, Yanfeng Qu, Jie Hao, Suyuan You, Bingsheng Li

**Affiliations:** 1 China Institute for Radiation Protection, Taiyuan 030006, China; linhpcirp@126.com (H.L.); 418192673@163.com (Y.Q.); Haoj@163.com (J.H.); 2 School of Nuclear Science and Technology, Lanzhou University, Lanzhou 730000, China; yousy19@lzu.edu.cn; 3 State Key Laboratory for Environment-Friendly Energy Materials, Southwest University of Science and Technology, Mianyang 621010, China

**Keywords:** silicon carbide, H_2_^+^ implantation, He^+^ implantation, bubbles, microstructure

## Abstract

Silicon carbide (SiC) is a promising material used in the advanced semiconductor industry. Fabricating SiC-on-insulator via H implantation is a good method. He and H co-implantation into Si can efficiently enhance exfoliation efficiency compared to only H implantation. In this study, 6H-SiC single crystals were implanted with He^+^ and H_2_^+^ dual beams at room temperature, followed by annealing at 1100 °C for 15 min, and irradiations with 60 keV He ions with a fluence of 1.5 × 10^16^ ions/cm^−2^ or 5.0 × 10^16^ ions/cm^−2^ and 100 keV H_2_^+^ ions with a fluence of 5 × 10^16^ ions/cm^−2^ were carried out. The lattice disorder was characterized by both Raman spectroscopy and transmission electron microscopy. The intensity of Raman peaks decreased with increasing fluence. No Raman shift or new phases were found. A very high numerical density of bubbles was observed as compared to single H or He implantation. Moreover, stacking faults, Frank loops and tangled dislocations were formed in the damaged layer. Surface exfoliation was inhibited by co-implantation. A possible reason for this is an increase in fracture toughness and a decrease in elastic out-of-plane strain due to dense bubbles and stacking faults.

## 1. Introduction

Due to its excellent physical, electronic and optical properties [1], SiC is considered as the main candidate material for next-generation large-scale integrated circuits. There are over 200 polytypes, but the hexagonal 4H- and 6H-SiCs are mainly considered for their advanced physical performances, such as the wide bandgap energy of 3 eV for 6H-SiC and 3.2 eV for 4H-SiC, low leakage currents and high operation temperatures. It is well known that SiC-on-insulator (SiCOI) structures have been widely considered due to their excellent properties, i.e., the low-power dissipation to save energy and the high radiation resistance to apply in space [2,3]. SiCOI structures can be fabricated by “smart-cut” technology, which was first reported by Bruel in 1995 [4]. There are three main processes of “smart-cut” technology. The first step is keV-MeV H ion implantation with a fluence of the order of 10^16^ to 10^17^ cm^−2^ at room temperature (10^16^ cm^−2^ for Si, GaAs or SiC, and 10^17^ cm^−2^ for GaN and ZnO) [5,6,7,8,9,10]. The second step is wafer bonding to another rigid substrate (handling wafer) before thermal annealing. Finally, due to fracturing, a thin layer transfer can be achieved at high temperatures. The critical factor is to form micro-cracks inside the sample. H-implantation-induced platelets cause the formation of micro-cracks. It has been reported that platelets consist of vacancy–hydrogen compounds. The detailed descriptions were illustrated in [11]. When the implanted depth is less than 1 μm, the growth of micro-cracks inside the Si, SiC, GaN or other wafers can induce surface blisters or exfoliation if the wafer is not bonded to a stiffer substrate. Tong et al. argued that the same activation energy can result in blister formation and layer splitting [12]. Therefore, there are many reports that have investigated the smart-cut threshold condition by means of the formation and growth process of surface blisters and exfoliation. Since surface blisters can reach micro-scales, they are easily observed by optical microscopy. Activation energy of blister growth with temperature or time can be obtained by in situ optical observation. It has been reported that H and He co-implantation can efficiently reduce the total fluence for Si wafer exfoliation. Agarwal et al. [13] reported that the implantation of both 1 × 10^16^ He/cm^2^ and a 7.5 × 10^15^ H/cm^2^ are sufficient to transfer a thin silicon film after 750 °C annealing compared to 5 ×10^16^ H/cm^2^ for single H implantation. Weldon et al. [14] reported that He plays a physical role as a source of internal pressure, leading to the reconversion of molecular H_2_ to bound Si-H type defects, which are precursory to the formation of platelets. The implantation order also affects exfoliation efficiency. Duo et al. argued [15] that it is efficient to exfoliate surfaces in samples implanted with hydrogen first. On the contrary, Nguyen et al. [16] considered that it can significantly enhance damage production during the first H implantation, leading to suppression of exfoliation. This is consistent with report of Daghbouj et al. [17] that whole He atoms participate in the pressurization of H-related platelets when He is implanted first, so micro-cracks grow quickly. Similarly, Radu et al. [18] reported low-temperature layer splitting of GaAs by He + H co-implantation and direct wafer bonding. A thin GaAs layer is transferred onto Si via a spin-on glass intermediate layer followed by annealing at 225 °C for 14 h.

What about He + H co-implantation into SiC? Would the similar functioning of He + H co-implantation enhance exfoliation in SiC? Shen et al. studied the surface morphology and microstructure of 6H-SiC irradiated by H and He ions [19]. They found blisters and exfoliation on the surface after irradiation. The bubble density in the samples irradiated by He and H is larger than that observed with single-ion irradiation, which would be attributed to the wide distribution of H nano-cracks in the vertical direction and the decrease in elastic modulus of the irradiated layer caused by He implantation. Bai et al. [20] studied the microstructure and mechanical properties of 6H-SiC irradiated by He and H ions. They found that He and H ion irradiation caused lattice deformation, and the lattice disorder recovered slightly after annealing, indicating that H ion irradiation retards the recovery of lattice defects. In addition, H ion irradiation may hinder the increase in hardness during annealing. It is clear that He ion irradiation plays a primary role in the damage process. Taguchi et al. studied the synergistic effect of helium and hydrogen injection on the microstructure of SiC–SiC composites [21]. He bubbles were formed in the irradiated samples, and the average size of He bubbles in the matrix decreased with increasing implanted H ion density. The numerical density of He bubbles increases with increasing hydrogen fluence. So far, the interaction between He bubbles and H_2_ molecules has not been fully evaluated. It is worth further investigating bubble formation after He and H_2_ co-implantation.

In this study, co-implantation with He and H_2_ ions was performed to study exfoliation efficiency after 1100 °C annealing. Lower exfoliation efficiency was found in the He and H co-implantation as compared to the single H implantation. Microstructure characterization presented a high density of bubbles instead of the micro-cracks formed after H implantation, which can explain the inhibition of surface exfoliation in the He and H co-implanted SiC.

## 2. Experiment

The single crystal 6H-SiC samples with [0001] crystal were supplied by HF-Kejing Inc. (Hefei, China). The sample was mirror polished with a size of 10 × 10 mm^2^ and a thickness of 0.3 mm. Hydrogen and helium implantation experiments were carried out on the 320 kV high charge ion integrated research platform of the Institute of Modern Physics, Chinese Academy of Sciences (Lanzhou, China). Helium and hydrogen molecular beams with energies of 60 keV and 100 keV, respectively, were chosen to implant samples close to the surface normal direction at room temperature. Electrical scanning was employed to obtain a uniform implantation with an area of 16 × 17 mm^2^, which is larger than the sample surface. The vacuum in the target chamber was lower than 5.0 × 10^−4^ Pa. He^+^ pre-implantation followed by setting H_2_^+^ at room temperature was performed. The helium fluence was 1.5 × 10^16^ ions/cm^2^ or 5 × 10^16^ ions/cm^2^ (hereafter references are made to “low” and “high” fluence samples, respectively), and the hydrogen molecular fluence was 5 × 10^16^ ions/cm^2^. SRIM 2013 software (Detailed Calculation with full Damage Cascades www.srim.org) was used to simulate the displacements per atom (dpa) and implanted ion concentration of H_2_^+^ and He^+^. The density used in the calculation was 3.21 g/cm^3^ and the displacement energies of C and Si were 20 eV and 35 eV [22], respectively. Post-implantation thermal annealing was performed at 1100 °C for 15 min in a vacuum (10^−3^ Pa).

Raman spectroscopy and transmission electron microscopy were carried out to study the lattice damage induced by H_2_^+^ and He^+^ implantation. Raman spectroscopy is a good method to avoid sample destruction during characterization which has been widely used to investigate ions implanted in SiC via the decrease in Raman scattering intensity, frequency shifting and broadening of the phonon Raman bands [23,24,25]. Confocal Raman scattering spectra were acquired at room temperature with a French HR-800 spectrometer in backscattering geometry with a 532 nm excitation laser radiation line. The spectral resolution was approximately 0.5 cm^−1^, and the grating was 600 lines/mm. The acquisition time of each spectrum was 30 s. The measured spectral range was 200 to 1000 cm^−1^. It is known that the absorption coefficient of single crystal 6H-SiC is 115 cm^−1^ at 532 nm [23]. Therefore, the penetration depth of excitation wavelength in single crystal 6H-SiC reaches 43 μm (d = 1/2λ, λ is the absorption coefficient), which is far larger than the penetration depth of H_2_^+^ and He^+^ ions. The microstructural evolution of the He- and H-induced damage was studied by cross-sectional transmission electron microscopy (XTEM) and high-resolution transmission electron microscopy (HRTEM) using a Tecnai G20 operated at 200 kV. Lattice defects were observed under the bright field and weak-beam dark field ((***g***, 5***g***), ***g*** = 0002). Bubbles were analyzed under kinetic imaging and out-of-focus conditions.

## 3. Results and Discussion

**Figure 1:**
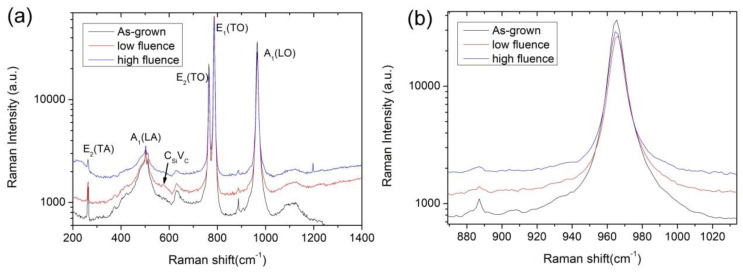
(**a**) Raman spectra of 6H-SiC irradiated with He and H_2_ ions at a low fluence (1.5 × 10^16^ He^+^/cm^2^ + 5 × 10^16^ H_2_^+^/cm^2^) or a high fluence (5 × 10^16^ He^+^/cm^2^ + 5 × 10^16^ H_2_^+^/cm^2^) at RT (Room temperature) followed by 1100 °C annealing for 15 min. (**b**) Zoom-in on A_1_(LO) peaks at different fluence conditions.

As the implantation fluence is larger than the threshold condition for amorphous transition, the typical Raman peaks of 6H-SiC disappeared (not shown). After 1100 °C annealing for 15 min, the recrystallization of material occurred, and Raman peaks became visible. Figure 1a presents the Raman spectra of 6H-SiC implanted with 60 keV He^+^ and 100 keV H_2_^+^ at room temperature and then annealed at 1100 °C for 15 min. Group theory selection rules predict that A_1_, E_1_ and E_2_ are the active modes in Raman spectra [24]. These are further divided into longitudinal (LO), transverse (TO) optical modes, longitudinal (LA) and transverse (TA) acoustical models. The first-order Raman bands of E_2_ (TO) at 767 cm^−1^, E_1_(TO) at 789 cm^−1^ and A_1_(LO) at 967 cm^−1^, as well as second-order Raman bands attributed E_2_(TA) at 266 cm^−1^, and A_1_(LA) at 504^−1^ cm^−1^ and 513 cm^−1^ were clearly observed. This indicates a good crystalline structure after recrystallization. The baseline intensity increased with increasing fluence, which is related to Rayleigh scattering from the implantation-induced defects. Normally, the A_l_(LO) peak is regarded as a reference for damage level. Figure 1b shows the A_l_(LO) peaks within the 880–1020 cm^−1^ range at different fluence conditions. One can see that the integral intensities of the A_1_(LO) Raman peaks decreased with the increase in He fluence, while there was no shift in the peak positions, indicating the recovery of lattice strain after thermal annealing. High-resolution XRD can also confirm the decrease in strain intensity with increasing annealing temperature [26]. The Raman intensity is related to the quality of crystalline, and the decrease in Raman intensity with increasing absorption coefficients could be attributed to the implantation-induced lattice defects. From Figure 1a, we can see the existence of one broad peak at 578 cm^−1^ in the low and high fluence samples, which is obvious in the low fluence sample. This phonon peak stemmed from the vibration mode of C_Si_V_C_ complexes [27]. Density function theory simulation confirmed that the activation energy of the defect recombination process ranges from 0.22 to 1.6 eV for C_Si_V_C_, indicating that it can be stable at 1100 °C [28].

**Figure 2:**
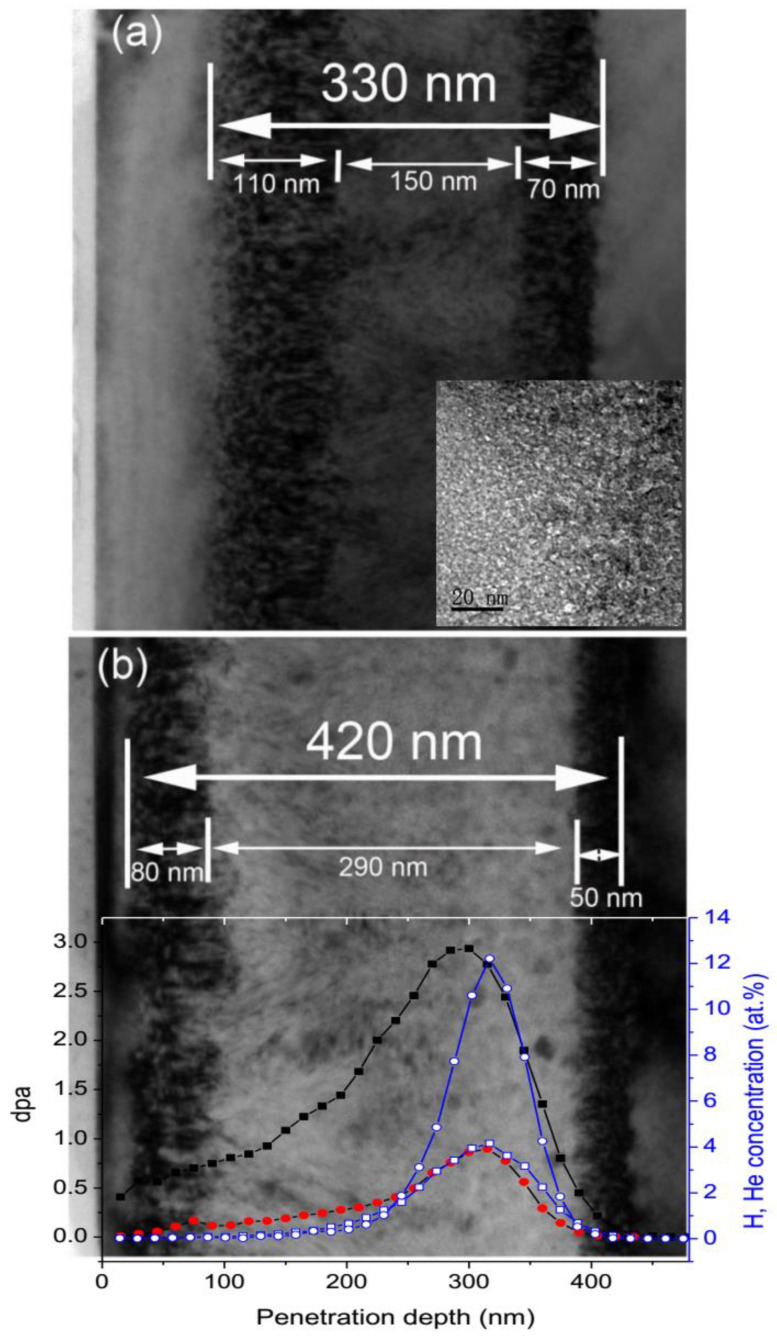
(**a**) XTEM bright-field micrographs of 6H-SiC implanted with the low fluence. (**b**) The high fluence, followed by annealing at 1100 °C for 15 min. Inset in (**a**) taken from the middle of the damaged layer was observed under the under-focused condition. In (**b**) are superposed with SRIM-2013 simulation the profiles of irradiation damages (solid) and atom concentrations (open) (squares for He implantation at a fluence of 5 × 10^16^ He^+^/cm^2^ and circles for H_2_^+^ implantation at a fluence of 5 × 10^16^ H_2_^+^/cm^2^).

The depth distributions of lattice defects after He and H implantation are shown in Figure 2. In parallel, the profiles of irradiation damage and atom deposition concentration simulated by SRIM-2013 overlap in Figure 2a. There is a well-defined defect band beneath the sample. The contrast in the damaged zone arises from the lattice strains of various lattice defects. The width of the damaged band has 330 nm at the low fluence and 420 nm at the high fluence. The damaged band can be divided into three layers, two black-contrasted layers with thicknesses of ~110 and ~70 nm, respectively, and a bright-contracted layer in the middle with a thickness of ~150 nm. The black contrasts come from Bragg diffraction, corresponding to extended defects formed after annealing. Many bubbles were visible in the bright-contrasted layer which coincide with the maximum H and He concentrations, as shown in the inset in Figure 2a. In Figure 2b, we can see that the bubble layer becomes wider with increasing fluence. In front of the damaged band, the extended defect layer is wider than the end of the damaged layer. The distribution of the observed lattice disorder is consistent with the profiles of simulated curves.

**Figure 3:**
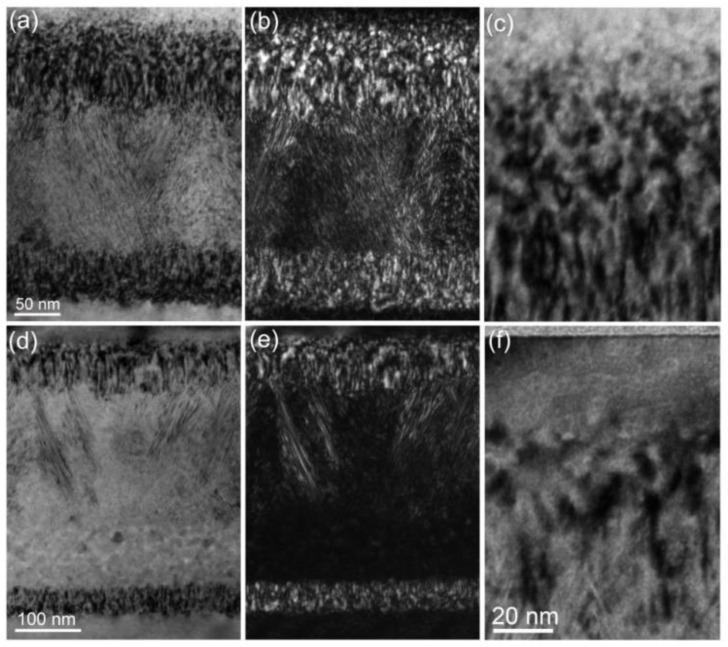
XTEM microstructural images of the damaged band observed under (**a**,**d**) bright field and (**b**,**e**) weak-beam dark field ((g, 5g), g = 0002) (a and b for the low fluence, d and e for the high fluence), (**c**,**f**) magnified images taken from the upper damage band of (**a**,**d**), respectively. The sample surface is vertical and upright.

The lattice defects were studied by weak-beam bright-field and dark-field microscopy with (g, 5g), g = 0002, as shown in Figure 3. Under bright-field imaging conditions, defects exhibit black contrast and the opposite is the case under dark-field imaging conditions. Similar to Figure 2, the damage region can be divided into three different bands (upper, middle and bottom bands). Near the surface region, we can see that there are many Frank loops with b = 1/2<0001> and tangled dislocations with b = 1/6<2$\bar{2}$03>, as shown in Figure 3c,f. One can see both the concentrations and sizes of these defects in the low fluence condition are larger than those in the high fluence condition. Meanwhile, in Figure 3a,b, we can see that the width of the middle damaged band increases with increasing fluence. The upper damaged band trended to the specimen surface, resulting in some lattice defects that were easily trapped by the surface. This is understandable as the ion energy is in the order of keV; the surface trapping effect on defect evolution becomes significant [29]. In addition, from Figure 3, we can see that more bubbles would be formed with increasing fluence, and these bubbles can also retard the growth of tangled dislocations [30].

**Figure 4:**
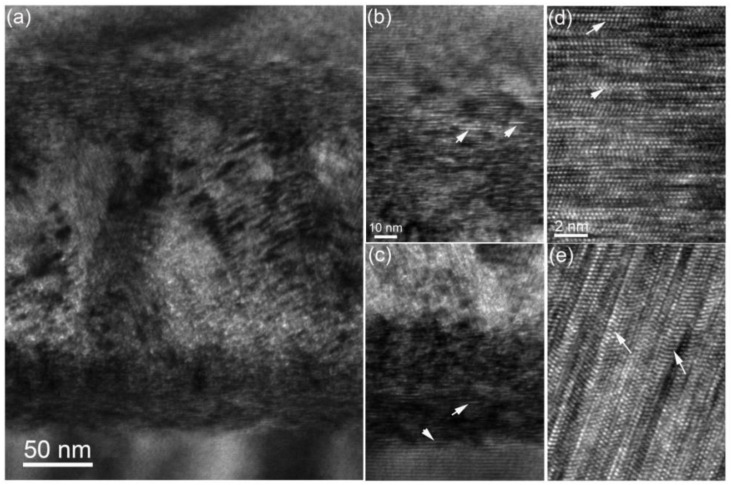
(**a**) Bright-field XTEM images under two-beam (g = 1$\bar{1}$00) showing the damaged band in the 6H-SiC after the low fluence implantation. High-resolution images of (**b**,**d**) in front of the damaged band showing stacking faults indicated by white arrows, (**c**) below the damaged band and (**e**) in the middle of the damaged band, showing columnar grains with many stacking faults.

In order to investigate the morphology of the damaged layer carefully, high-magnification XTEM images were analyzed, as shown in Figure 4. From Figure 4, we can see that many stacking faults along the plane of the specimen surface were observed under the two-beam condition with **g** = 1$\bar{1}$00. This is reasonable when considering the low stacking fault energy in SiC, which leads to the easy formation of stacking faults in the high-temperature thermal annealing process. The growth of stacking faults can produce twin boundaries, which would improve the fracture toughness and radiation resistance of SiC [31]. Besides that, columnar grains and bubbles were also detected in the middle of the damaged band. The observed lattice defects were similar to those of He-implanted SiC at RT followed by high-temperature annealing [32].

**Figure 5:**
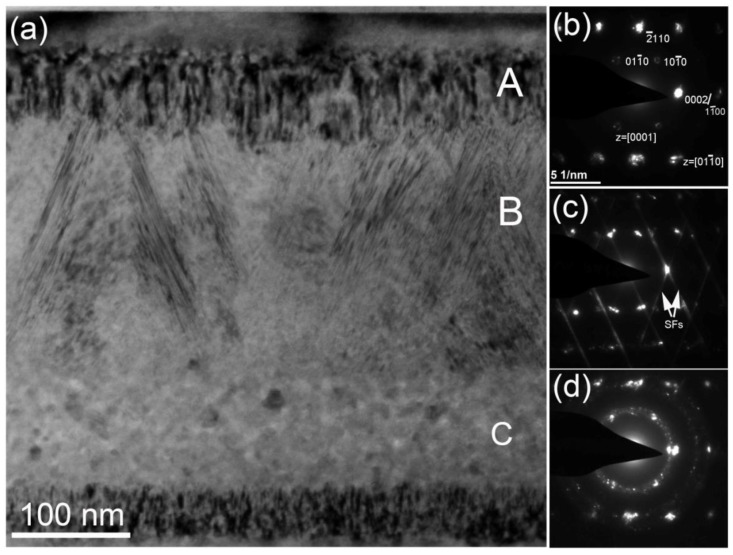
(**a**) Bright-field XTEM image of SiC implanted with the high fluence. (**b**–**d**) show selected area electron diffraction patterns taken from the A, B and C zones, respectively.

Selected area electron diffraction was performed to measure grain structure after co-implantation. In the A zone, many tangled dislocations were formed. The diffraction pattern confirmed two different grains. One grain has bright diffraction spots and the axis is [01$\bar{1}$0], originating from the substrate. The other has weak diffraction spots and the axis is [0001], which was observed after recrystallization of He-implanted SiC at RT [33]. In the B zone, the diffraction spots have long rods, originating from stacking faults in the columnar grain (see Figure 4e). In addition, twin boundaries from 3C-SiC were found in Figure 5c. In the C zone, the arc of the diffraction spots was observed, indicating polycrystalline structures in this region. Meanwhile, compared with other zones, there were high densities of bubbles in the C zone, which may suggest that the existence of bubbles can retard recrystallization growth, consistent with our recent report [30].

**Figure 6:**
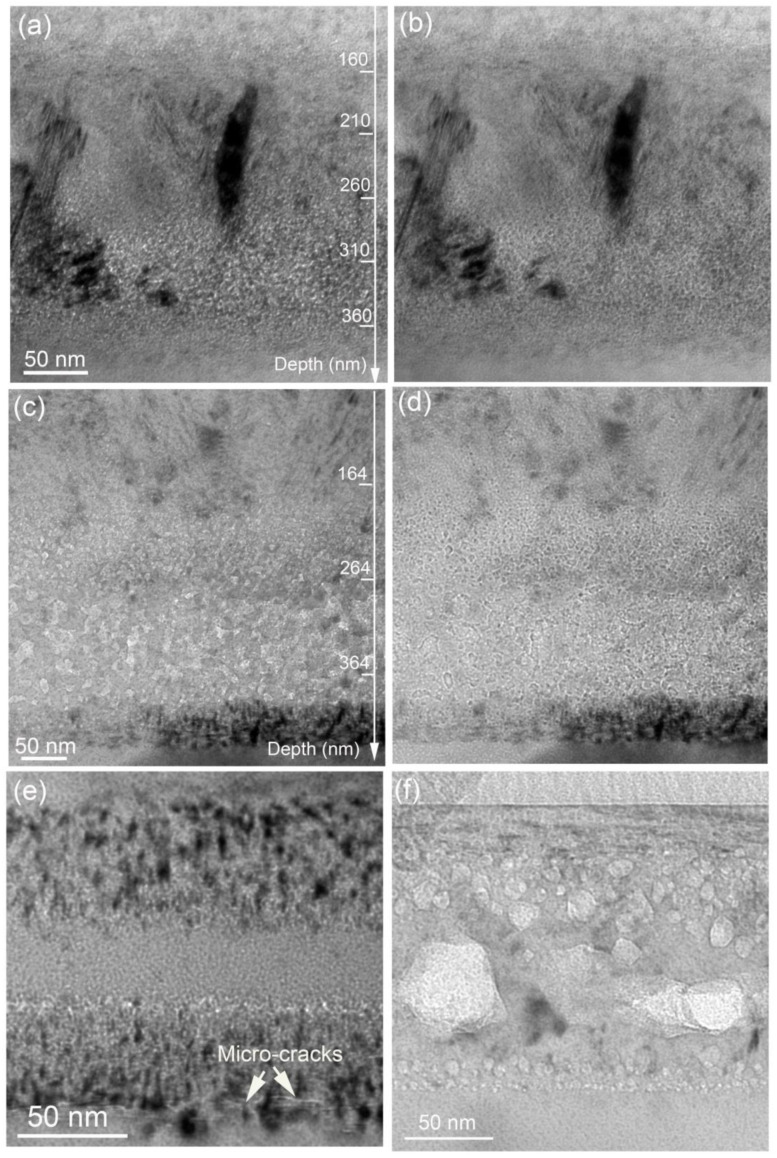
Bubble formation in He and H_2_ co-implanted 6H-SiC with the low fluence: (**a**) under-focus, (**b**) over-focus; (**c**–**e**) high fluence 134 keV H_2_^+^ implantation at a fluence of 5 × 10^16^/cm^2^ followed by 1100 °C annealing for 15 min; (**f**) 15 keV He^+^ implantation at a fluence of 1 × 10^17^/cm^2^ followed by 1200 °C annealing for 30 min.

Bubbles can be confirmed with defocus conditions, exhibiting Fresnel contrast, as shown in Figure 6a,b. For the low fluence implantation, a bubble layer at the depth ranging from 110 nm to 380 nm can be seen. The observed bubbles that have a number density on the order of 10^23^/m^3^ are only several nanometers in diameter. With increasing fluence, the bubble layer becomes wider, from 100 nm to 440 nm. The shape of some bubbles becomes polygonal, similar to He-implanted SiC at a fluence of 4.4 × 10^17^/cm^2^ followed by 900 °C annealing for 30 min [34]. By comparison, nano-sized bubbles and micro-cracks were formed in the H_2_^+^-implanted SiC at a fluence of 5 × 10^16^/cm^2^ followed by 1100 °C annealing for 15 min, as shown in Figure 6e. Surface exfoliation and blisters can be observed [6]. However, large, faceted cavities with a size near 50 nm can be observed in the He^+^ implantation at a fluence of 1 × 10^17^/cm^2^ followed by 1200 °C annealing for 30 min, as shown in Figure 6f. In this case, the surface becomes even and flat [30].

Compared with the separated implantation in Figure 6e,f, we can see that He and H co-implantation can result in an increase in bubble density but a decrease in bubble size. In the present experiment, He^+^ ions were pre-implanted, resulting in the formation of interstitials and vacancies in the sample. Subsequently, H_2_^+^ ions were implanted. He atoms and H atoms can occupy interstitial sites and vacancy sites. According to the binding energies calculated by Sun et al., He and H prefer to accumulate into vacancies [35]. After 1100 °C annealing, gas–vacancy clusters grew into nano-sized bubbles based on the Oswald ripening mechanism [36]. Vacancy clusters hardly migrate when they contain H_2_ molecular and He atoms [37]. Moreover, the recombination of vacancies and interstitials becomes difficult once these vacancies contain gas. Thermal desorption spectrometry confirmed that there are two desorption peaks, one at 600 K and the other at 1200 K for 1 keV He implantation into SiC [38]. The low temperature is attributed to interstitial He atoms and the high temperature is assigned to He–vacancy clusters. More He–vacancy clusters can be formed with the increase in He fluence, resulting in the shift of desorption temperature to a higher value. In comparison with single He implantation, He and H co-implantation produced more vacancies to form He–vacancy clusters. When considering the high binding energy of He atoms with bubbles [39], it is reasonable to regard He desorption from bubbles as becoming more difficult in the He and H co-implanted SiC than in the single He implantation. Therefore, in the single He implantation bubbles are large and faceted after 1200 °C annealing, but in He and H co-implantation many nano-sized bubbles are formed after 1100 °C annealing. These results suggest that irradiation swelling in the He and H co-implantation condition could be smaller than that in the single He implantation condition.

For the single H_2_ implantation followed by 1100 °C annealing, only dispersed bubbles 1–2 nm in diameter were observed, these being much smaller than bubbles formed after He and H co-implantation. This may be due to the larger concentration of vacancies available in the He pre-implanted SiC which facilitates the nucleation and growth of bubbles during annealing. Some micro-cracks aligning on a (0001) plane were formed at the end of the damaged band (see Figure 6e), corresponding to the occurrence of surface exfoliation [6]. However, in the co-implantation condition, surface blistering was inhibited. Matani and Gosele [40] developed a model for the onset of blistering:

(1) $$r_{cric}=\left\{\frac{16\gamma Et^{3}}{9\alpha \left(1-v^{2}\right)\Delta p^{2}}\right\}1/4$$

where Δ*p* is the difference between the inside the platelets and the outside atmosphere, *t* is the microcrack depth, *E* is the material’s Young’s modulus, *v* is Poisson’s ratio, α is a numerical factor in the order of ~1, and *γ* is the specific interface energy which would increase by the implantation-induced defects. Wang et al. [41] calculated diffusion behaviors of H in the He pre-implanted SiC, and they found the energy barrier for H migration up to 0.95 eV compared to 0.50 eV in the pure SiC. These results suggest that the He pre-implantation can retard the diffusion of H in SiC, resulting in a decrease in the inner pressure of bubbles. At the same time, there are more co-implantation-induced vacancies that act as bubble nucleations to reduce the gas density in bubbles. Therefore, the critical radius for the onset of blistering increases significanly in the He and H co-implanted 6H-SiC. This demonstrates that surface blisters become difficult with increasing lattice disorder. Moreover, because He atom mass is two times as large as H_2_ molecular mass, cascade collision is higher for He implantation [42]. Hochbauer et al. [43] investigated the ion-cut in H-implanted Si with different fluences, and they reported a rapid decrease in the elastic out-of-plane strain and an increase in fracture toughness in heavily damaged Si. The formation of platelets became difficult in the heavily damaged region, so less surface blistering occurred at a fluence of 1 × 10^17^/cm^2^. The same mechanism can be responsible for SiC; exfoliation efficiency can decrease to zero when the implantation fluence is larger than the threshold fluence (3 × 10^16^/cm^2^–1 × 10^17^/cm^2^). SiC is an important nuclear structural material, and the low value of the fracture toughness limits its application. It has been reported that lattice defects, such as twin boundaries, can enhance the fracture toughness of ceramics [44].

Unlike the increased exfoliation efficiency in Si co-implanted with He and H, the co-implantation of He and H in SiC can decrease its exfoliation efficiency. The detailed reason could be that lattice defects can easily move and anneal out in Si, and therefore He implantation-induced lattice defects have less influence on the growth of platelets. Meanwhile, He atoms have a smaller migration energy in Si than in SiC (0.96 eV vs. 1.5 eV), resulting in He atoms that migrate quickly and accumulate into H_2_ gas bubbles and increase the inner pressure. It has been reported that the increase in the inner pressure can enhance the growth of platelets into micro-cracks [14]. We performed He implantation with a fluence of 8 × 10^15^/cm^2^ + H implantation with a fluence of 1 × 10^16^/cm^2^ at RT followed by annealing at 1100 °C for 15 min and 30 min. No surface blisters or exfoliation could be found [45]. Therefore, we see synergy efficiency in Si co-implanted with He and H ions, but not in SiC.

## 4. Conclusions

In this study, the formation and evolution of lattice damage in 6H-SiC co-implanted with He^+^ and H_2_^+^ followed by 1100 °C annealing for 15 min were studied by Raman spectroscopy and transmission electron microscopy. The main conclusions are as follows:

1. Raman spectra show that defects produced by ion implantation are not completely recovered after high-temperature annealing at 1100 °C for 15 min. This is due to the bubbles formed during annealing, which retard the recovery of lattice damage. The increase in ion fluence leads to a decrease in crystallization peak intensity.
2. Microstructure observation shows recrystallization during annealing at 1100 °C. In the damaged band, bubbles, dislocation loops and stacking faults are formed, similar to single He implantation. In comparison with single H_2_ or He implantation, an increase in bubble density but a decrease in bubble size in the He and H co-implantation is observed.
3. Surface exfoliation is retarded by He and H_2_ co-implantation because He implantation-induced lattice defects inhibit the formation of micro-cracks in the damaged band.

## Data Availability

The data presented in this study are available on request from the corresponding authors.
